# The Impact of Obesity on Perioperative Complications in Prolapse Patients ≥ 70 Years Old

**DOI:** 10.1007/s00192-026-06747-9

**Published:** 2026-06-10

**Authors:** Stephanie W. Zuo, Rachel Noorani, Kristina Warner, Anne M. Suskind, Halina M. Zyczynski, Mary F. Ackenbom

**Affiliations:** 1 Division of Urogynecology and Reconstructive Pelvic Surgery, Department of Obstetrics and Gynecology, University of Virginia School of Medicine, Charlottesville, VA 22902 USA; 2 Division of Urogynecology and Reconstructive Pelvic Surgery, Department of Obstetrics and Gynecology, University of Pittsburgh Medical Center and Magee-Womens Research Institute, Pittsburgh, PA USA; 3 University of Virginia School of Medicine, Charlottesville, VA USA; 4 Division of Urogynecology and Reconstructive Pelvic Surgery, Department of Obstetrics and Gynecology, John H. Stroger, Jr. Hospital of Cook County, Chicago, IL USA; 5 Department of Urology, University of California, San Francisco, CA USA; 6 Division of Urogynecology and Reconstructive Pelvic Surgery, Department of Obstetrics and Gynecology, University of Michigan, Ann Arbor, MI USA

**Keywords:** Obesity, Older adult, Postoperative outcomes, Prolapse surgery, Surgical complications

## Abstract

**Introduction and Hypothesis:**

Obesity is a recognized risk factor for developing pelvic organ prolapse and for perioperative complications. As the population ages, an increasing number of older, obese individuals with prolapse may opt for surgical repair. This study assesses the association between obesity and short-term perioperative adverse events (AEs) in women ≥ 70 years undergoing vaginal and laparoscopic prolapse surgery.

**Methods:**

This is a secondary analysis of a retrospective cohort study of older women undergoing vaginal and laparoscopic prolapse surgery at two medical centers from 2016 to 2023. Patients were classified as nonobese (< 30 kg/m^2^), class 1 obese (30–34.9 kg/m^2^), or class 2+ obese (≥ 35 kg/m^2^). The primary outcome was composite AEs within 8 weeks after surgery, and secondary outcomes included major AEs and individual AEs.

**Results:**

Of 1507 older women, 20.8% (*n* = 314) were class 1 obese and 6.0% (*n* = 90) were class 2+ obese. Obese patients were younger and more likely to be frail, hypertensive, and diabetic (*p* < 0.05). Obese patients experienced greater estimated intraoperative blood loss (*p* < 0.05). Obesity was not associated with composite AEs or major AEs on multivariable analysis, but was found to be predictive of postoperative hematoma. Class 1 obese women had a 2.7-times greater odds (95% CI 1.17–6.22) and class 2+ obese women had a 4.7-times greater odds (95% CI 1.61–13.43) of hematoma compared to nonobese women. Obesity was not associated with major hematoma, defined as ≥5 cm or requiring medical or surgical intervention.

**Conclusions:**

Obesity in older surgical patients is not associated with a greater incidence of composite AEs, but may be associated with postoperative hematomas.

**Supplementary Information:**

The online version contains supplementary material available at https://doi.org/10.1007/s00192-026-06747-9.

## Introduction

Obesity and older age in women are both risk factors for developing pelvic organ prolapse. Rates of obesity in the United States are high, with estimates close to 45%, and women are more likely to be obese than men [1]. In addition, the population of adults aged 70 years and older is rising. By the year 2030, one in five Americans will be retirement age, and given this, the number of women experiencing bothersome prolapse is expected to reach nearly 5 million [2]. Surgical repair of prolapse is a highly desirable option for many patients. One in eight women undergoes surgery for prolapse by the age of 80 [3]. Given these trends, reconstructive pelvic surgeons should expect to see an increasing number of obese, older women desiring surgery for pelvic organ prolapse.

There is limited data on the impact of obesity on procedure-related complications for older patients undergoing prolapse surgery. In other surgical specialties such as orthopedics, obese patients aged 65 years and older have a significantly increased risk of wound infection and dehiscence [4, 5]. On the other hand, for endometrial cancer patients undergoing minimally invasive surgery, cohort studies suggest that obesity does not impact complication rates [6]. In prolapse surgery, several retrospective studies have demonstrated similar rates of perioperative adverse events (AEs) in nonobese and obese individuals regardless of severity of obesity [7–9]. However, the average age of obese patients in these studies was below 60 years. Further research is needed on the synergistic impact of obesity and older age on prolapse surgery outcomes.

The aim of this study is to compare perioperative AEs within 8 weeks after prolapse surgery between nonobese, class 1 obese, and class 2+ obese women aged 70 years and older. We hypothesized that class 1 and 2+ obese women aged ≥ 70 years would be more likely to experience AEs in the perioperative period compared to nonobese women. Results of this study can guide clinicians with perioperative risk stratification, surgical decision-making, and perioperative counseling for older, obese patients with prolapse.

## Materials and Methods

This was a secondary analysis of a dual-center retrospective cohort study of women 61 years of age and older undergoing pelvic organ prolapse surgery by board-certified urogynecologists from January 2016 to May 2023. The original study examined the impact of perioperative pharmacologic thromboprophylaxis on wound and bleeding complications in high-risk patients undergoing prolapse surgery. In this study, which was conceptualized after completion of the original study, only patients ≥ 70 years of age with recorded body mass index (BMI) values were included. An age cutoff of 70 years was chosen as the peak incidence of care-seeking for pelvic floor disorders occur in the 70–79 year age range, yet few studies focus on outcomes in this age group and older [10]. Patients were grouped by obesity category: nonobese (< 30 kg/m^2^), class 1 obese (30–34.9 kg/m^2^), or class 2+ obese (≥ 35 kg/m^2^). Class 2 (35–39.9 kg/m^2^) and class 3 (40 kg/m^2^) obese patients were merged into a single group due to low numbers of class 3 obese patients in the cohort. Prolapse surgeries included obliterative surgery (colpocleisis and colpectomy), both apical and non-apical vaginal repairs (uterosacral ligament suspension, sacrospinous ligament suspension, isolated anterior and/or posterior colporrhaphy, transvaginal mesh), and minimally invasive abdominal prolapse repair (robotic and laparoscopic sacrocolpopexy and uterosacral ligament suspension). Patients undergoing open surgery for prolapse were excluded.

The primary outcome was a composite AE outcome inclusive of perioperative complications within 8 weeks after surgery. AEs were collected by extensive chart review, which included available outside records. The composite AE outcome included intraoperative complications (conversion to laparotomy, bladder injury, ureteral injury, bowel injury, and estimated blood loss (EBL) ≥ 500 mL) and postoperative complications, which included reoperation, hospital readmission, emergency room evaluation, discharge to skilled nursing facility, cardiac complication (any cardiac event that required cardiologist consultation, e.g., myocardial infarction, atrial fibrillation), venous thromboembolism, stroke or transient ischemic attack, blood product transfusion, hematoma (diagnosed clinically or by imaging and categorized into major, defined as ≥ 5 cm and/or requiring medical or surgical intervention, and minor hematoma, defined as < 5 cm in size, expectantly managed, or managed with minor office procedure such as cauterization), ileus or small bowel obstruction, surgical site infections, wound dehiscence, urinary tract infection, death, and other complications (defined as adverse events that did not fall into the previously listed categories). A time frame of 8 weeks postoperative was used given that the majority of complications after pelvic organ prolapse surgery occur within the first 2 months [11]. A planned sub-analysis for a composite major AE outcome was performed which excluded urinary tract infections, superficial surgical site infections, and other complications. Individual AEs were also compared by obesity category.

Clinical and demographic variables and perioperative AEs were collected through both automated and manual extraction of the electronic medical record. These included age, parity, smoking status, race and ethnicity, preoperative pelvic organ prolapse quantification (POP-Q) stage, relevant medical comorbidities and Charlson Comorbidity Index score (a measure of multimorbidity), frailty as defined by the 5-modified Frailty Index, prior hysterectomy and reconstructive pelvic surgery, prolapse surgery characteristics, including type, concomitant procedures, anesthesia approach, length of surgery, estimated blood loss, and same-day hospital discharge. Independent variables were compared between obesity categories using chi-square (or Fisher’s exact) analyses and analyses of variance (ANOVA) or Kruskal–Wallis tests if nonparametric. Multivariable logistic regression models were developed to control for variables that were chosen a priori based on their clinical relevance (age, hypertension, diabetes, frailty, minimally invasive abdominal approach, concomitant hysterectomy, and concomitant incontinence procedure) when assessing for the association between obesity and composite and major AEs. Statistical analyses were performed using STATA/SE 17.0 (StataCorp LLC, College Station, TX, USA).

## Results

The study cohort included 1507 women undergoing prolapse repair with a mean age of 76.2 ± 5.0 years and a mean BMI of 27.4 ± 4.6 kg/m^2^. Of these women, 314 (20.8%) were class 1 obese and 90 (6.0%) were class 2+ obese. Table 1 provides a summary of the demographic, clinical, and surgical characteristics of the cohort. Compared to nonobese patients, obese patients were younger, but more likely to be frail on the 5-item Modified Frailty Index (28.4% class 2+ obese vs 19.2% class 1 obese vs 13.1% nonobese, *p* < 0.01). Patients had comparable multimorbidity by Charlson Comorbidity Index score and similar preoperative prolapse severity. Obese women were more likely to have had a prior hysterectomy (53.3% class 2+ obese vs 46.3% class 1 obese vs 40.6% nonobese, *p* = 0.02). There were no differences in the type of prolapse surgery except for isolated colporrhaphy (anterior and/or posterior) which was more common in obese patients (27.8% class 2+ obese vs 20.4% class 1 obese vs 16.4% nonobese, *p* = 0.01). Intraoperatively, obese patients experienced greater EBL (median 75 mL class 2+ obese vs 50 mL class 1 obese vs 50 mL nonobese, *p* = 0.01), despite similar rates of concomitant hysterectomy and incontinence surgery, as well as comparable operative times.

**Table 1:** Patient demographic and clinical characteristics

	Nonobese (*n* = 1103)	Class 1 obese (*n* = 314)	Class 2+ obese (*n* = 90)	*p* value
Age, years	76.5 (5.1)	75.2 (4.4)	75.0 (4.2)	** <0.01**
Body mass index, kg/m^2^	25.3 (2.8)	31.9 (1.4)	38.3 (3.2)	** <0.01**
Parity	2 (2–3)	3 (2–4)	3 (2–4)	0.11
Current smoker	32 (3.0)	4 (1.3)	1 (1.2)	0.21
Hispanic ethnicity	5 (0.5)	4 (1.3)	0 (0)	0.23
Race				0.24
White	1055 (97.4)	294 (95.8)	84 (96.6)	
Black	17 (1.6)	10 (3.3)	3 (3.5)	
Other	11 (1.0)	3 (1.0)	0 (0)	
Preoperative pelvic organ prolapse quantification stage				0.07
Stage 2	244 (22.4)	66 (21.0)	23 (25.6)	
Stage 3	700 (64.2)	191 (61.0)	48 (53.3)	
Stage 4	147 (13.5)	55 (17.8)	19 (21.1)	
Past medical history				
Hypertension	632 (58.6)	215 (68.7)	66 (75.0)	** <0.01**
Diabetes	142 (13.2)	65 (20.8)	18 (20.5)	** <0.01**
History of cancer	235 (21.8)	65 (20.8)	18 (20.5)	0.91
Use of Chronic anticoagulation	95 (8.7)	34 (10.9)	13 (14.4)	0.13
Charlson Comorbidity Index	4 (3–5)	4 (3–5)	4 (3–5)	0.60
Frail status (score 2+ on 5-mFI)	141 (13.1)	60 (19.2)	25 (28.4)	** <0.01**
Past surgical history				
Prior hysterectomy	447 (40.6)	145 (46.3)	48 (53.3)	**0.02**
Prior prolapse surgery	153 (13.9)	51 (16.2)	18 (20.0)	0.21
Prior incontinence surgery	73 (6.7)	26 (8.3)	8 (8.9)	0.50
Surgery type				
Sacrocolpopexy	165 (15.0)	43 (13.7)	7 (7.8)	0.16
Vaginal apical suspension (native tissue)*	281 (25.5)	68 (21.7)	18 (20.0)	0.23
Colpectomy/colpocleisis	492 (44.6)	146 (46.5)	41 (45.6)	0.83
Isolated colporrhaphy (anterior and/or posterior)	181 (16.4)	64 (20.4)	25 (27.8)	**0.01**
Transvaginal prolapse mesh	5 (0.5)	1 (0.4)	0 (0)	1.00
Surgical approach				0.27
Vaginal approach	892 (80.9)	255 (81.2)	79 (87.8)	
Minimally invasive abdominal approach	211 (19.1)	59 (18.8)	11 (12.2)	
Other surgical characteristics				
Concomitant hysterectomy	435 (39.4)	120 (38.2)	28 (31.1)	0.29
Concomitant incontinence procedure	78 (7.1)	30 (9.6)	11 (12.2)	0.10
General anesthesia	864 (94.4)	229 (95.0)	73 (96.1)	0.80
Length of surgery, min	126.6 (62.2)	129.8 (69.0)	121.8 (67.1)	0.74
Estimated blood loss, mL	50 (30–100)	50 (30–100)	75 (45–200)	**0.01**
Discharge home with prophylactic anticoagulation	77 (10.4)	24 (11.3)	7 (12.5)	0.84
Same-day hospital discharge	800 (72.5)	211 (67.2)	60 (66.7)	0.12

Data are presented as mean (standard deviation), median (interquartile range), or *n* (%). Bolded values are significant (*p* < 0.05)

*5-mFI: *5-item Modified Frailty Index

^*^Vaginal apical suspension with native tissue includes uterosacral ligament and sacrospinous ligament suspension

The incidence of composite AEs did not differ by obesity class (27.0% nonobese, 28.7% class 1 obese, 31.1% in class 2+ obese, *p* = 0.63) (Table 2). Major AEs, which excluded postoperative urinary tract infections, superficial surgical site infections, and other complications, also did not differ by obesity class (14.0% nonobese, 11.8% class 1 obese, 17.8% class 2+ obese, *p* = 0.32). The only AE that differed by obesity class was postoperative hematoma with an incidence of 1.2% in nonobese patients versus 3.2% in class 1 obese patients vs 5.6% in class 2+ obese patients (*p* < 0.01). On multivariable logistic regression controlling for use of chronic anticoagulation and concomitant hysterectomy, class 1 obese women had a 2.7-times greater odds (95% CI 1.17–6.22) and class 2+ obese women had a 4.7-times greater odds (95% CI 1.61–13.43) of hematoma compared to nonobese women. Notably, major hematoma incidence did not differ by obesity class (*p* = 0.48). Details on hematoma location and management by obesity class, as well as incidence of hematoma by surgical approach and type, are listed in Supplemental Tables 1 and 2.

**Table 2:** Adverse events by obesity status

	Nonobese (*n* = 1103)	Class 1 obese (*n* = 314)	Class 2+ obese (*n* = 90)	*p* value
Composite adverse events	298 (27.0)	90 (28.7)	28 (31.1)	0.63
Major adverse events*	154 (14.0)	37 (11.8)	16 (17.8)	0.32
Intraoperative complications				
Conversion to open approach	2 (0.2)	2 (0.6)	0 (0)	0.39
Urinary tract injury	5 (0.5)	2 (0.6)	1 (1.1)	0.41
Bowel injury	1 (0.1)	0 (0)	0 (0)	1.00
EBL ≥500 mL	2 (0.2)	2 (0.8)	0 (0)	0.37
Postoperative complications				
Death	1 (0.1)	0 (0)	0 (0)	1.00
Return to operating room	13 (1.2)	5 (1.6)	0 (0)	0.57
Readmission	37 (3.4)	8 (2.6)	3 (3.3)	0.80
ED evaluation	91 (8.3)	17 (5.4)	7 (7.8)	0.25
Discharge to SNF	6 (0.5)	2 (0.6)	1 (1.1)	0.45
Cardiac complication	8 (0.7)	1 (0.3)	1 (1.1)	0.49
Stroke/TIA	1 (0.1)	0 (0)	1 (1.1)	0.16
Venous thromboembolism	8 (0.7)	4 (1.3)	0 (0)	0.48
Transfusion	7 (0.6)	3 (1.0)	0 (0)	0.71
Hematoma	13 (1.2)	10 (3.2)	5 (5.6)	** <0.01**
Major hematoma†	9 (0.8)	5 (1.6)	2 (2.2)	0.48
Wound dehiscence	8 (0.7)	2 (0.6)	0 (0)	1.00
Ileus/small bowel obstruction	8 (0.7)	0 (0)	0 (0)	0.32
Postoperative UTI	130 (11.8)	32 (10.2)	12 (13.3)	0.64
Other complications**˚**	85 (7.9)	32 (10.3)	7 (8.1)	0.41
Surgical site infection	17 (1.5)	7 (2.2)	1 (1.1)	0.67
Superficial SSI	8 (0.7)	6 (1.9)	1 (1.1)	0.14
Deep SSI	5 (0.5)	0 (0)	0 (0)	0.70
Organ-space SSI	4 (0.4)	1 (0.3)	0 (0)	1.00

Data expressed in *n* (%); *ED *emergency department, *SNF *skilled nursing facility, *TIA *transient ischemic attack, *UTI *urinary tract infection, *EBL *estimated blood loss, *SSI *surgical site infection

^*^Excludes UTI, superficial SSI, and other complications

^†^Defined as hematoma ≥5 cm and/or requiring medical or surgical management

˚Other complications included: streptococcal pharyngitis, upper respiratory infection, sinusitis, bronchitis, pneumonia, diverticulitis, C. difficile infection, vulvovaginal candidiasis, skin candidiasis, thrush, COPD exacerbation, shingles, urinary retention requiring sling lysis, pudendal neuralgia, lower extremity neuralgia from positioning, postoperative cognitive dysfunction, fall, varicose vein bleeding, gastritis causing melena, fecal impaction

Bolded values are significant (*p* < 0.05)

On multivariable analysis controlling for age, hypertension, diabetes, frailty, minimally invasive abdominal approach, concomitant hysterectomy, and concomitant incontinence procedure, there remained no association between composite or major AEs and obesity class (Table 3). However, minimally invasive abdominal approach was found to have a 59% lower adjusted odds (95% CI 0.42–0.83) of composite AEs and a 56% lower adjusted odds (95% CI 0.35–0.91) of major AEs (Table 4). Age was associated with a higher odds of major AE, but not composite AE (adjusted OR 1.03, 95% CI 1.00–1.06).

**Table 3:** Multivariable analysis of association of obesity on adverse events

	Class 1 obesity†		Class 2+ obesity†	
	Unadjusted OR [95% CI]	Adjusted OR [95% CI]	Unadjusted OR [95% CI]	Adjusted OR [95% CI]
Composite AE*	1.09 [0.82–1.43]	1.05 [0.79–1.40]	1.22 [0.77 −1.94]	1.13 [0.70–1.83]
Major AE*	0.82 [0.56–1.21]	0.82 [0.56–1.22]	1.33 [0.76–2.35]	1.22 [0.67–2.21]
Hematoma˚	2.76 [1.20–6.36]	2.70 [1.17–6.22]	4.92 [1.71–14.14]	4.65 [1.61–13.43]

*OR *Odds ratio, *CI *Confidence interval, *AE *Adverse events

^†^Compared to nonobese

^*^Multivariable analyses for Composite AE and Major AE controlled for age, hypertension, diabetes, frailty, minimally invasive abdominal approach, concomitant hysterectomy, and concomitant incontinence procedure

**˚**Multivariable analyses for hematoma controlled for chronic anticoagulant use and concomitant hysterectomy

**Table 4:** Multivariable analysis of association of obesity and relevant covariates on composite and major adverse events

Composite AE	Adjusted OR [95% CI]
Class 1 obesity	1.05 [0.79–1.40]
Class 2+ obesity	1.13 [0.70–1.83]
Age	1.02 [0.99–1.04]
Hypertension	1.22 [0.94–1.59]
Diabetes	1.38 [0.84–2.26]
Frail status by mFI-5	0.98 [0.59–1.64]
Minimally invasive abdominal approach	0.59 [0.42–0.83]*
Concomitant hysterectomy	1.09 [0.85–1.40]
Concomitant incontinence procedure	0.95 [0.62–1.46]
Major AE	Adjusted OR [95% CI]
Class 1 obesity	0.82 [0.56–1.22]
Class 2+ obesity	1.22 [0.67–2.21]
Age	1.03 [1.00–1.06]*
Hypertension	1.26 [0.90–1.78]
Diabetes	1.06 [0.56–2.02]
Frail status by mFI-5	1.17 [0.61–2.24]
Minimally invasive abdominal approach	0.56 [0.35–0.91]*
Concomitant hysterectomy	1.18 [0.86–1.63]
Concomitant incontinence procedure	0.72 [0.39–1.32]

*OR *Odds ratio, *CI *Confidence interval, *AE *Adverse events

*Significance defined as *p* < 0.05

## Discussion

Our analyses of obese, older women undergoing pelvic organ prolapse surgery did not identify an increased risk of perioperative complications compared to nonobese women. Obese women in this cohort were younger, but paradoxically more likely to be frail, have hypertension, and diabetes compared to nonobese women. We found that obese, older patients are at an elevated risk for developing postoperative hematomas and are more likely to have greater estimated blood loss at the time of surgery, despite undergoing similar surgical approaches. Hematomas ranged from minor hematomas managed conservatively (*n* = 12, 42.9%), to major hematomas at least 5 cm in size and/or requiring antibiotics for suprainfection or surgical intervention by the primary surgeon or interventional radiology (*n* = 16, 57.1%). A minority of major hematomas (*n* = 3/28, 10.7%) required blood transfusion, and none of these occurred in class 2+ obese patients. Overall, rates of major AEs were low across all groups, with the most common AEs being urinary tract infections and other minor complications.

Our study adds to the literature on outcomes after prolapse surgery in older patients. The baseline characteristics of our cohort mirrors existing studies. For example, the incidence of obesity in our cohort (26.9%) is comparable to prior published studies on prolapse surgery outcomes which report incidence rates ranging from 17.8–34.4% in populations that include younger patients [7–9, 12, 13]. In addition, the incidence of complications in our study is similar to prior work in older adults ≥ 65 years undergoing prolapse surgery where the composite outcome includes minor complications (14, 15). In studies of older patients using American databases, such as the American College of Surgeons National Surgical Quality Improvement Program, overall complication rates after prolapse surgery are lower, which may be due to shorter timeframe of follow-up (30 days) and a limited set of complications that does not include emergency room evaluation and other minor complications [13].

In this older cohort, there was no difference in composite or major AEs by obesity class, even when adjusting for confounding variables. This is largely consistent with other studies on perioperative complications in younger, obese women undergoing prolapse repair. Multiple studies, including a recent meta-analysis on the effect of obesity on sacrocolopopexy outcomes, have detected no significant differences in perioperative complication rates by obesity class after accounting for differences in surgery type and comorbidities [7, 9, 13, 16]. Even morbidly obese patients (BMI ≥ 40 kg/m^2^) undergoing vaginal apical prolapse repairs have comparable rates of 30-day complications compared to patients with normal weight [8, 17]. On the other hand, one US study suggested that laparoscopic prolapse surgery in morbidly obese patients is associated with a higher incidence of major complications [17]. This is in contrast to our cohort which identified a decreased odds of complications in patients who underwent a minimally invasive abdominal approach for prolapse repair. This finding may be due to surgeon selection bias favoring healthier patients as well as a surgically optimized patient cohort undergoing minimally invasive abdominal prolapse surgery. Another retrospective, single-center cohort study of women undergoing vaginal urogynecologic surgery found that obese patients had a 5.5-times greater adjusted odds of experiencing surgical site infections [18]. There was no increased rate of infection or major complications with increasing obesity in our cohort. Our contrasting findings should be considered in light of the cohort having few morbidly obese patients (*n* = 21, 1.4%) and a significant proportion of obliterative prolapse repairs (45%), which is to be expected in an older patient population and may be associated with fewer surgical site infections [19].

Although composite AEs were not increased in obese patients, class 2+ obese patients had greater median EBL, and both class 1 and class 2+ obese patients were more likely to develop postoperative hematomas. There is evidence that increasing BMI is associated with higher EBL during gynecologic surgery. In a single-center study of patients undergoing minimally invasive sacrocolpopexy, obesity was associated with greater blood loss compared to overweight women (mean 82.4 mL vs 63.8 mL) [20]. Hadizadeh et al. found that morbidly obese patients undergoing apical prolapse repair had greater blood loss compared to normal weight patients (mean 233.6 mL vs 115.2 mL) [8]. On the other hand, existing studies on both prolapse surgeries and hysterectomy have reported no increased incidence of perioperative hematomas in obese compared to nonobese patients [18, 21]. Most hematomas in our study occurred after vaginal prolapse surgery (*n* = 27, 96.4%), with the majority occurring after an obliterative procedure (*n* = 18, 64.3%). It is possible that obesity could increase the risk of hematomas due to greater pressure on pelvic incisions during healing and/or increased adipose tissue which could predispose to subcutaneous bleeding. Notably, the incidence of major hematomas and their potential downstream effects (e.g., excessive bleeding requiring transfusion, surgical site infections, wound dehiscence) did not differ by obesity class, which suggests that the elevated hematoma risk detected in class 1 and 2+ obese patients may not significantly impact clinical outcomes. Further research is needed to verify these findings using larger cohorts and to examine whether the increased hematoma risk affects other outcomes, such as healthcare utilization or patient recovery experiences.

Strengths of this study include its inclusion of patients from two medical centers, focus on an older patient population aged 70 years and above, and detailed review of AEs up to 8 weeks after prolapse surgery. Multivariable models were developed to more accurately examine the impact of obesity on prolapse surgery outcomes. There are however some limitations intrinsic to secondary analyses that should be considered when interpreting study findings. Our cohort had low racial diversity with 95.1% of the cohort identifying as White race. Power calculations were not performed for individual AEs, and therefore, these findings should be considered as hypothesis-generating. Additionally, a relatively low proportion of class 2 obese patients and even fewer class 3 obese patients resulted in grouping of class 2 and 3 obesity patients, which limits applicability of findings for morbidly obese patients. In other words, readers should not assume that associations related to class 2+ obese patients in this study directly apply to morbidly obese patients. Lastly, the database did not include long-term complications or patient-reported outcomes, which could have furthered our understanding of the impact of obesity in older adults undergoing prolapse surgery.

In conclusion, obesity does not increase overall complication rates after prolapse surgery in women 70 years and older. The elective nature of surgical repair for prolapse and the ability to optimize patients preoperatively may account for the comparable rates of AEs across obesity classes in this older cohort. Reconstructive pelvic surgeons should be reassured that surgical management of prolapse can be offered to appropriate older, obese patients, thus increasing the opportunity for quality-of-life improvement in a population which might not routinely be considered ideal for surgery.

## Supplementary Information

Below is the link to the electronic supplementary material.

Supplementary file1 (DOCX 17 KB)

## Data Availability

The data that support the findings of this study are available on request from the corresponding author.
