# Supplementary material for: The Impact of Obesity on Perioperative Complications in Prolapse Patients ≥ 70 Years Old
Source: Int Urogynecol J. 2026 Jun 10;37(10):3475–82. doi: 10.1007/s00192-026-06747-9 (PMC13731712; doi:10.1007/s00192-026-06747-9)
Supplement: Supplementary file 1 [file 192_2026_6747_MOESM1_ESM.docx]

| Supplemental Table 1: Clinical Details of Hematoma by Obesity Status | | | |
| --- | --- | --- | --- |
| **Location of Hematoma** | **Expectant management˚** | **Major hematoma*** | **Transfusion** |
| ***Non-obese patients*** | | | |
| Distal vagina |  |  |  |
| Distal vagina | X |  |  |
| Distal vagina | X |  |  |
| Distal vagina | X |  |  |
| Distal vagina | X | X |  |
| Distal vagina |  | X |  |
| Distal vagina |  | X |  |
| Perineal | X | X |  |
| Pelvic |  | X |  |
| Pelvic | X | X |  |
| Pelvic |  | X | X |
| Abdominal (laparoscopic port site) |  | X |  |
| Presacral | X | X |  |
| ***Class 1 obese patients*** | | | |
| Distal vagina | X |  |  |
| Distal vagina |  |  |  |
| Perineal | X |  |  |
| Pelvic/vaginal cuff |  | X | X |
| Pelvic |  | X |  |
| Pelvic | X | X |  |
| Abdominal (laparoscopic port site) | X |  |  |
| Retroperitoneal |  | X | X |
| Not specified | X |  |  |
| Not specified | X |  |  |
| ***Class 2+ obese patients*** | | | |
| Distal vagina |  |  |  |
| Labial | X |  |  |
| Vaginal cuff |  | X |  |
| Vagina (intraoperative) |  |  |  |
| Pelvic | X |  |  |
| ˚ Expectantly managed hematomas were observed and required no intervention (excludes patients who required office cauterization)  *Major hematoma defined as ≥5cm in size and/or required surgical or medication interventions (i.e., oral or intravenous antibiotics, reoperation, procedures including drainage or embolization). | | | |

| Supplemental Table 2: Hematoma by Surgical Approach and Type | | |
| --- | --- | --- |
|  | **Hematoma**  **n=28** | **p-value** |
| **Surgical Approach** | | 0.04* |
| **Vaginal approach** | 27 (96.4) |  |
| **Minimally invasive abdominal approach** | 1 (3.6) |  |
| **Surgery Type** | | |
| **Sacrocolpopexy** | 1 (3.6) |  |
| **Vaginal apical suspension (native tissue)*** | 3 (10.7) |  |
| **Colpectomy/Colpocleisis** | 18 (64.3) |  |
| **Isolated Colporrhaphy (Anterior and/or Posterior)** | 6 (21.4) |  |
| **Transvaginal prolapse mesh** | 0 (0) |  |
| Data presented as n (%). | |  |
